# Central Nervous System Prophylaxis Approach in High‐Risk Diffuse Large B‐Cell Lymphoma Patients: A Retrospectively Collected, Single‐Center Cohort Analysis

**DOI:** 10.1002/cam4.70830

**Published:** 2025-04-01

**Authors:** Yanli Wang, Xiaolian Wen, Tao Guan, Hongwei Zhang, Wei'e Han, Min Bai, Xiaolan Liu, Min Zhang, Liping Su, Weihua Zhang

**Affiliations:** ^1^ Shanxi Medical University Affiliated Cancer Hospital/Shanxi Provincial Cancer Hospital/Chinese Academy of Medical Sciences Cancer Hospital Affiliated Shanxi Hospital Hematology Department Taiyuan China; ^2^ First Hospital of Shanxi Medical University Taiyuan China

**Keywords:** CNS prophylaxis, CNS relapse, diffuse large B‐cell lymphoma, systemic high‐dose methotrexate

## Abstract

**Objectives:**

Recent advances in the prevention of diffuse large B‐cell lymphoma (DLBCL) have considerably focused on optimal strategies for preventing its recurrence in the central nervous system (CNS) in patients. This retrospective study aimed to assess the protective efficacy of intravenous high‐dose methotrexate (HD‐MTX) regimens in newly diagnosed patients with DLBCL presenting a high risk for CNS recurrence.

**Methods:**

A total of 136 newly diagnosed high‐risk DLBCL patients (HD‐MTX group: *n* = 46; non‐HD‐MTX group: *n* = 90) were enrolled in this retrospective study. The primary endpoints included CNS recurrence rate, progression‐free survival (PFS), overall survival (OS), and toxicity.

**Results:**

The 2‐year CNS recurrence rates (median follow‐up period: 25.5 months; 95% confidence interval: 21.0–30.0) were 4.3% and 11.1% in the HD‐MTX and non‐HD‐MTX groups (*p* = 0.337), respectively. Additionally, the 2‐year progression‐free survival (PFS) and overall survival (OS) rates were 70.7% versus 60.8% and 72.9% versus 60.8% (*p* = 0.013 and *p* = 0.024), respectively. The subgroup analysis for PFS and OS revealed that patients classified as the National Comprehensive Cancer Network (NCCN)—International Prognostic Index (IPI) low‐ or intermediate‐risk, at a younger age, and without B symptoms demonstrated potential benefits from the HD‐MTX treatment. In total, 46 patients completed 92 cycles of HD‐MTX treatment, of which, 49 cycles were administered on day 6 of the R‐CHOP regimen, with an average delay of no more than 4 days. In contrast, the remaining 43 cycles were initiated on days 10–14 following the completion of the R‐CHOP regimen, with an average delay of 15 days. Interestingly, the incidence of hematological or non‐hematological toxicity did not differ significantly among the groups.

**Conclusion:**

Owing to the lack of robust evidence, the role of HD‐MTX in preventing CNS recurrence could not be conclusively determined. Nevertheless, some patients could tolerate the treatment, such as younger individuals and those at NCCN‐IPI low or intermediate risk, suggesting the efficacy of intravenous HD‐MTX administration on day 6 as an optimal strategy for preventing CNS recurrence of DLBCL.

**Abbreviations**
ASCT: autologous stem cell transplantation
CI: confidence interval
CNS: central nervous system
CSF: cerebral spinal fluid
DLBCL: diffuse large B‐cell lymphoma
GCB: Germinal center B‐cell
HD‐MTX: high‐dose methotrexate
HR: hazard ratio
IPI: International Prognostic Index
NCCN: National Comprehensive Cancer Network
NHL: non‐Hodgkin's lymphoma
OS: overall survival
PFS: progression free survival
R‐CHOP: rituximab, cyclophosphamide, doxorubicin, vincristine, and prednisone
TT‐CNS: the CNS recurrence time

## 1 Introduction

Diffuse large B‐cell lymphoma (DLBCL) has been reported to be the most prevalent subtype of non‐Hodgkin's lymphoma [1]. Patients with DLBCL are mainly treated with immunochemotherapy, such as the rituximab, cyclophosphamide, doxorubicin, vincristine, and prednisone (R‐CHOP) regimen. However, despite the treatment, 30%–40% of patients experience relapse or refractory disease, resulting in unfavorable prognoses [2]. Furthermore, approximately 2%–5% of patients with DLBCL have been reported to experience central nervous system (CNS) recurrence, with a median overall survival of 3.5 months following the onset of CNS recurrence [3, 4, 5].

The CNS International Prognostic Index (IPI) is the standard model for predicting the risk of CNS recurrence and incorporates both adrenal involvement points and IPI factors [4]. Patients with a CNS‐IPI score of 4–6 have been reported to present a CNS recurrence risk of > 10%. DLBCL incidence at high‐risk extranodal sites, such as the testes, kidneys, and breasts, can also result in a CNS recurrence risk of > 10% [6, 7]. Furthermore, patients with double‐hit lymphomas have been shown to exhibit a 13% incidence of CNS recurrence within 3 years [8]. Despite notable advances in identifying and mitigating the risk of CNS recurrence utilizing molecular‐typing methods, addressing financial implications and validation of reported methods in larger cohorts remain critical for their integration into standard clinical practice [9].

To reduce CNS recurrence, various treatment strategies have been developed to integrate CNS‐penetrating prophylaxis into frontline therapy. Notably, intravenous high‐dose methotrexate (HD‐MTX) is a commonly recommended CNS prophylaxis over intrathecal therapy, as most relapses are parenchymal in nature [10, 11, 12]. Many studies have reported enhanced survival outcomes following the incorporation of this CNS prophylaxis into the standard regimen [5, 13, 14]. However, recent studies have raised concerns regarding the efficacy of HD‐MTX [15, 16, 17]. Additionally, there remains a lack of consensus regarding the optimal delivery method for MTX, particularly in terms of timing.

Hence, this study aimed to assess the protective efficacy of HD‐MTX against CNS recurrence and its influence on survival outcomes in newly diagnosed patients with DLBCL, which presents a high risk for CNS recurrence. Additionally, different HD‐MTX treatment regimens and associated toxicities were investigated in this study.

## 2 Methods

### 2.1 Patient Enrollment

Herein, patients diagnosed with DLBCL between October 2018 and July 2023 were identified by retrospectively reviewing the registry dataset of the Shanxi Cancer Hospital. Patients presenting a high risk for CNS recurrence, who had received at least two cycles of standard R‐CHOP or R‐CHOP‐like chemotherapy, were enrolled in this study. The following criteria were used for assessing the high risk of CNS recurrence: (1) CNS‐IPI score ≥ 4; (2) involvement of organs such as the adrenal glands, kidneys, testes, and breast; and (3) fluorescence in situ hybridization results indicating double‐ or triple‐hit lymphoma. The following exclusion criteria were established for patients to minimize selection bias: (1) age ≥ 75 years, (2) baseline serum creatinine level ≥ 97 μmol/L, and (3) intolerance to HD‐MTX. Additionally, patient medical records, including baseline characteristics and relevant factors associated with the DLBCL recurrence rate, were collected and organized. Before treatment, individuals with CNS‐IPI score of ≥ 4 underwent cranial magnetic resonance imaging (MRI) and/or lumbar puncture; therefore, patients with either suspected or confirmed central infiltration at baseline assessment were excluded from this study.

### 2.2 Treatment and Response Assessment

The standard treatment protocols administered to patients were systematically organized and summarized, focusing on the initial targeted immunotherapy, number of cycles, and subsequent consolidation therapy. Patient responses were assessed bi‐cyclically, employing the Lugano classification to delineate tumor response. CNS involvement was confirmed based on either positive cerebral spinal fluid (CSF) conventional cytology, CSF flow cytometry, or biopsy. Additionally, recurrence was considered for those who presented clinical symptoms indicating CNS involvement and typical lesions on MRI.

### 2.3 CNS Prophylaxis

Intravenous HD‐MTX (recommended dosage: 3–3.5 g/m^2^) was administered as a prophylactic measure for patients presenting any of the three aforementioned risk factors. A review of HD‐MTX administration methods revealed that before 2019, HD‐MTX was predominantly administered between days 10 and 14 following conventional chemotherapy. However, since 2019, the initiation of administration has shifted to day 6 of conventional chemotherapy. Before initiating HD‐MTX administration, rigorous hydration and urine alkalinization were performed, and folic acid rescue was initiated after 12 h of HD‐MTX administration.

### 2.4 Statistical Analyses

Herein, the interval from the initiation of chemotherapy to CNS recurrence onset was defined as the CNS recurrence time (TT‐CNS), and the interval from chemotherapy initiation to disease progression, whichever occurs first, as progression‐free survival (PFS). The interval from chemotherapy initiation to death from any cause was defined as overall survival (OS). The Kaplan–Meier method was employed to estimate CNS relapse and survival rates, and survival curves across different groups were compared employing the log‐rank test. Univariate and multivariate analyses for CNS recurrence, PFS, and OS were conducted using the Cox proportional hazards regression model. Categorical variables were compared using Fisher's exact test or the chi‐square test, whereas Student's *t*‐test was used to evaluate continuous variables. All statistical analyses were performed using the GraphPad Prism 9 software, and the significance threshold was set at *p* < 0.05.

### 2.5 Ethics Declarations

All methods of CNS prophylaxis and chemotherapy regimens were conducted per the guidelines established by the National Comprehensive Cancer Network (NCCN) and the Chinese Society of Clinical Oncology for lymphoid malignancies. Informed consent was obtained from all patients and/or their legal guardians before their inclusion in the study. This study adhered to good clinical practice and the principles outlined in the Declaration of Helsinki. The methods of this study were approved by the Ethics Committee of the Shanxi Medical University Affiliated Cancer Hospital (Shanxi Provincial Cancer Hospital), which is registered as an Institutional Review Board with the National Medical Research Registration and Filing Information System (https://www.medicalresearch.org.cn) (approval number KY2023080).

## 3 Results

### 3.1 Patient Characteristics

**FIGURE 1:**
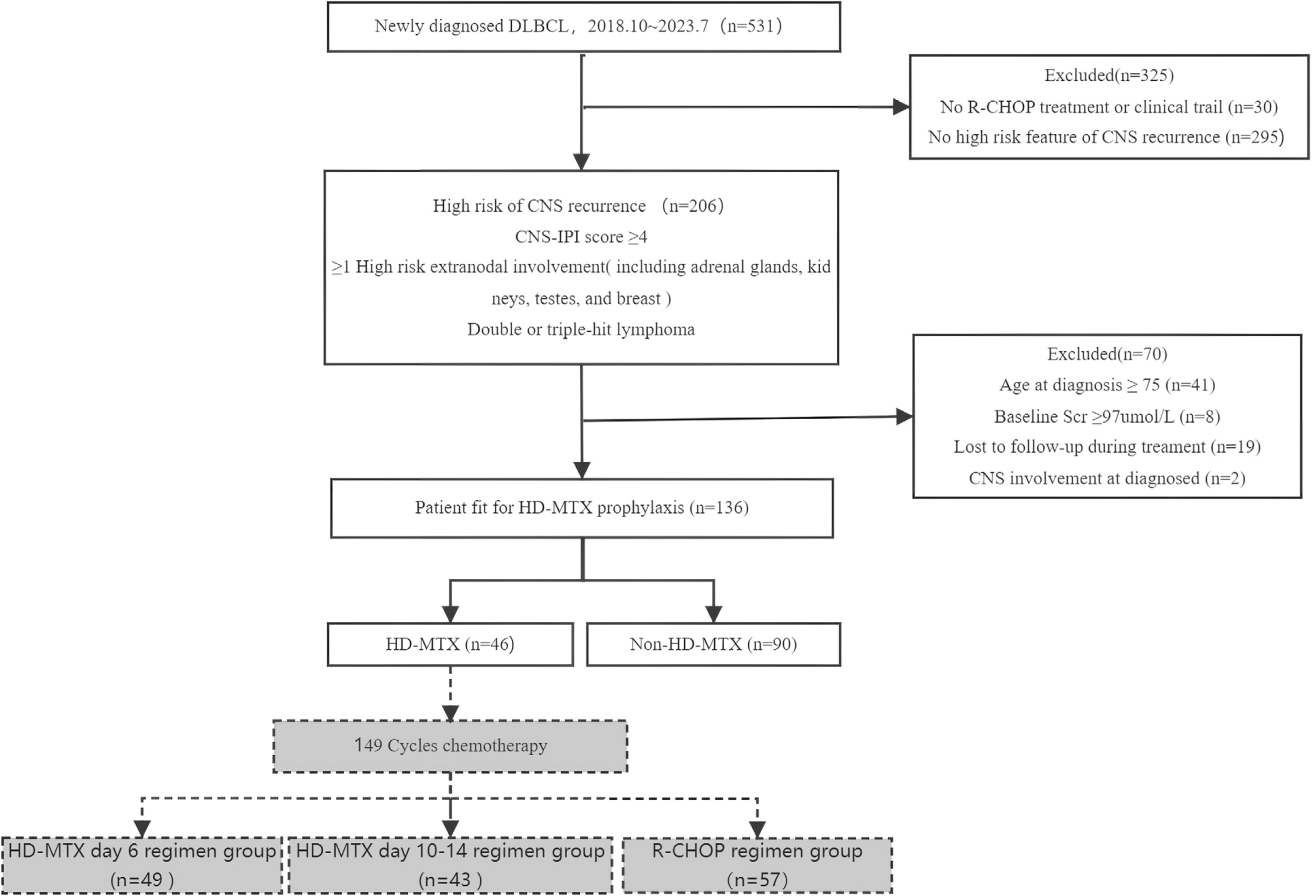
Consort flow diagram. SCr, serum creatinine. October 2018 to July 2023.

We identified 531 patients with newly diagnosed DLBCL during the study period. Of them, 206 patients were enrolled in this study, who were diagnosed with DLBCL (October 2018–July 2023) and met the high‐risk criteria for CNS recurrence (Figure 1). After excluding 49 patients owing to their excessive baseline creatinine levels and ≥ 75 years of age, 19 patients who were unable to adhere to follow‐up protocols, and 2 patients with pre‐existing CNS involvement, a total of 136 patients remained in the final cohort. They were then categorized into the following two groups: the non‐HD‐MTX group (*n* = 90) and the HD‐MTX group (*n* = 46). The baseline characteristics of the different patient groups are summarized in Table 1. Notably, both groups exhibited similarities regarding autologous stem cell transplantation (ASCT) status, extranodal involvement, Ann Arbor stage, NCCN‐IPI, and CNS‐IPI. However, a higher proportion of younger patients were present in the HD‐MTX group compared with that in the non‐HD‐MTX group.

**TABLE 1:** Baseline characteristics.

	HD‐MTX group (*n* = 46)	Non‐HD‐MTX group (*n* = 90)	All patients (*n* = 136)	*p*
Sex				
Female (%)	17 (37.0)	36 (40.0)	53 (39.0)	
Male (%)	29 (63.0)	54 (60.0)	83 (61.0)	0.731
Age				
Median (range), *y*	53.0 (20–73)	62.5 (19–74)	59.4 (19–74)	< 0.001^a^
Cell of origin				
GCB(%)	21 (45.7)	37 (41.1)	58 (42.6)	0.612
Non‐GCB(%)	25 (54.3)	53 (58.9)	78 (57.4)	
Double hit^b^				
Yes(%)	7 (7.8)	7 (7.8)	14 (10.3)	0.177
No (%)	39 (84.8)	83 (92.2)	122 (89.7)	
B symptoms				
Yes(%)	18 (39.1)	26 (28.9)	44 (32.4)	0.227
No(%)	28 (60.9)	64 (71.1)	92 (67.6)	
Ann Arbor stage				
I (%)	4 (8.7)	5 (5.6)	9 (6.6)	0.320
II (%)	5 (10.9)	5 (5.6)	10 (7.4)	
III (%)	3 (6.5)	11 (12.2)	14 (10.3)	
IV (%)	34 (73.9)	69 (76.7)	103 (75.7)	
NCCN‐IPI scores				
0 ~ 1 (%)	5 (10.9)	2 (2.2)	7 (5.1)	0.161
2 ~ 3 (%)	9 (19.6)	15 (16.7)	24 (17.6)	
4 ~ 5 (%)	22 (47.8)	52 (57.8)	74 (54.4)	
≥ 6 (%)	10 (21.7)	21 (23.3)	31 (22.8)	
CNS‐IPI scores				
≥ 4 (%)	28 (60.9)	69 (76.7)	97 (71.3)	0.054
< 4 (%)	18 (39.1)	21 (23.3)	39 (28.7)	
Intrathecal				
Yes (%)	13 (28.3)	15 (16.7)	28 (20.6)	0.114
No (%)	33 (71.7)	75 (83.3)	108 (79.4)	
Involvement of the kidney and adrenal gland				
Yes (%)	14 (30.4)	19 (21.1)	33 (24.3)	0.230
No (%)	32 (69.6)	71 (78.9)	103 (75.7)	
Involvement of the testis				
Yes (%)	10 (21.7)	9 (10.0)	19 (14.0)	0.062
No (%)	36 (78.3)	81 (90.0)	117 (86.0)	
Involvement of the breast				
Yes (%)	7 (15.2)	11 (12.2)	18 (13.2)	0.626
No (%)	39 (84.84)	79 (87.8)	118 (86.8)	
Autologous stem cell transplantation				
Yes (%)	10 (21.7)	11 (12.2)	21 (15.4)	0.146
No (%)	36 (78.3)	79 (87.8)	103 (83.7)	

Abbreviations: CNS, central nervous system; GCB, germinal center B‐cell; HD, high‐dose; IPI, International Prognostic Index; MTX, methotrexate; NCCN, National Comprehensive Cancer Network.

^a^ Represents statistical significance.

^b^ Denotes the presence of MYC translocation with B‐cell lymphoma (BCL)2 and/or BCL6.

Notably, in the HD‐MTX group, 24 patients received treatment before remission, 18 after remission, and 4 during progression. Additionally, 17 patients underwent one course, 13 underwent two courses, and 16 underwent three or more courses of HD‐MTX treatment.

### 3.2 CNS Recurrence

CNS recurrence was observed in 12 patients (8.7%) (median follow‐up period: 25.5 months; 95% confidence interval [CI]: 21.0–30.0), with 10 cases in the non‐HD‐MTX group and 2 cases in the HD‐MTX group. In these patients, TT‐CNS was 5.5 months (95% CI: 3.0–12.5). However, CNS relapse rates between the HD‐MTX and non‐HD‐MTX groups showed no significant differences, with a 2‐year cumulative incidence of 4.3% and 11.1%, respectively (*p* = 0.337).

Additionally, Cox regression survival analysis was conducted to identify major factors influencing CNS recurrence in enrolled patients presenting high risk for CNS recurrence. Univariate analysis revealed that a CNS‐IPI score of ≥ 4 (*p* = 0.018; hazard ratio [HR]: 7.022, 95% CI: 2.201–22.40) and the presence of B symptoms (*p* = 0.022; HR: 3.438, 95% CI: 0.987–11.970) were associated with an increased risk of CNS recurrence. However, multivariate analysis revealed no independent risk factors associated with CNS recurrence.

### 3.3 Survival Outcomes

**FIGURE 2:**
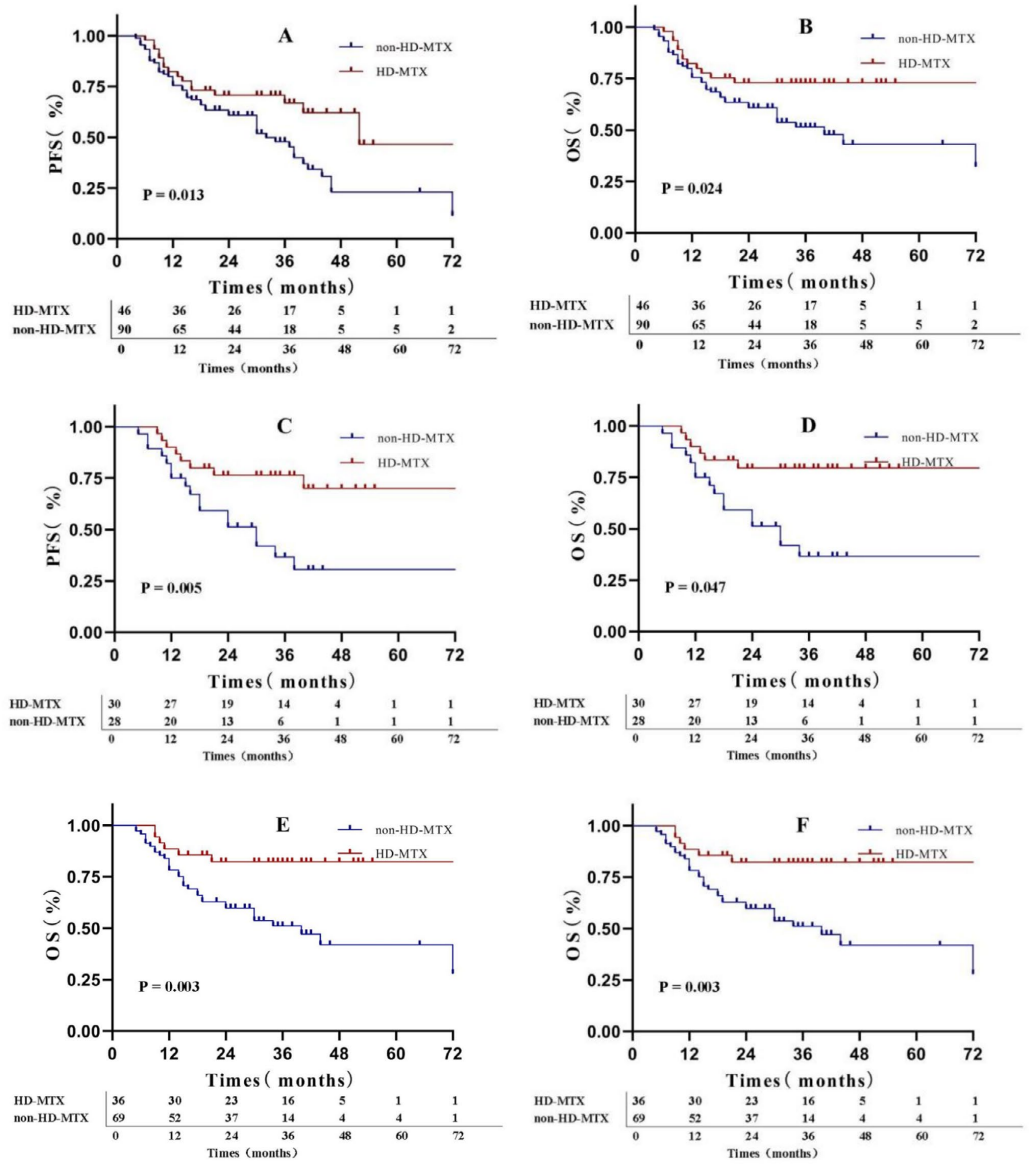
Survival outcomes of different central nervous system prophylaxis groups. (A) PFS and (B) OS in the overall population due to HD‐MTX prophylaxis (C) PFS and (D) OS in the younger population (age ≤ 60). (E) PFS and (F) OS in the National Comprehensive Cancer Network‐International Prognostic Index low‐ or intermediate‐risk population. The *X*‐axis unit is months and the *Y*‐axis unit is percentage. HD, high‐dose; MTX, methotrexate; OS, overall survival; PFS, progression‐free survival.

**FIGURE 3:**
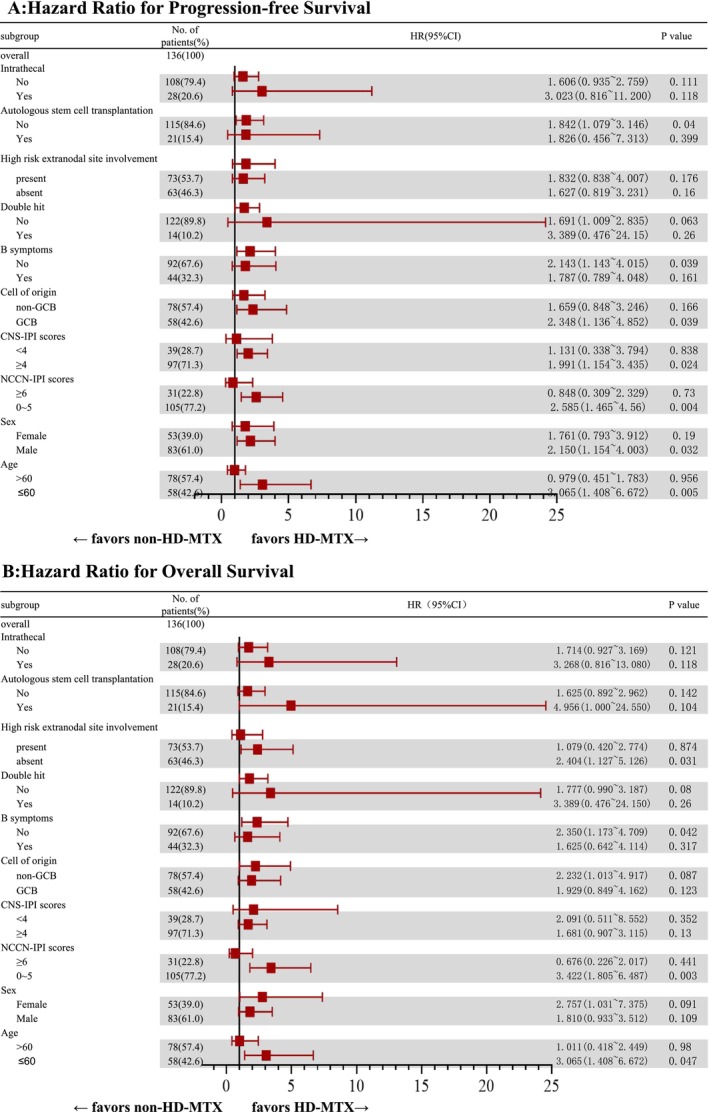
Forest plot for subgroup analyses. Hazard Ratios for (A) progression‐free survival and (B) overall survival. CNS, central nervous system; GCB, germinal center B‐cell; HD, high‐dose; IPI, International Prognostic Index; MTX, methotrexate; NCCN, National Comprehensive Cancer Network.

In the final patient cohort, the 2‐ and 5‐year PFS rates were 64.2% and 32.8%, respectively, whereas the 2‐ and 5‐year OS rates were 64.9% and 54.4%, respectively. The difference in OS and PFS was statistically significant between the HD‐MTX and non‐HD‐MTX groups (Figure 2A,B). Specifically, the 2‐year PFS and OS rates in the HD‐MTX group were 70.7% and 72.9%, respectively, whereas those in the non‐HD‐MTX group were 60.8% and 60.8%, respectively (for PFS, *p* = 0.013; HR: 0.506, 95% CI: 0.310–0.826; for OS, *p* = 0.024; HR: 0.490, 95% CI: 0.283–0.849). The results of subgroup analyses of PFS and OS indicated that patients classified as low‐ or intermediate‐risk patients per NCCN‐IPI, younger than 60 years of age, without B symptoms, who did not undergo ASCT, with germinal center B‐cell (GCB) type, with CNS‐IPI score ≥ 4, and of male sex showed greater benefit from the HD‐MTX treatment (Figure 2C–F and Figure 3A,B). The subgroup analysis was exploratory, with no preset assumptions, and the results should be interpreted with caution.

The results of Cox regression survival analysis revealed factors influencing PFS and OS in patients at high risk for CNS recurrence. Univariate analysis identified the following factors influencing PFS and OS: stage IV disease, CNS‐IPI scores ≥ 4, involvement of the testes or kidneys and adrenal glands, absence of CNS prophylaxis (intrathecal or HD‐MTX therapy), and CNS recurrence. Multivariate analysis revealed CNS‐IPI scores ≥ 4 and CNS recurrence as the significant factors (Table 2).

**TABLE 2:** Multivariate Cox regression analyses of risk factors for progression‐free and overall survival.

	PFS		OS	
	HR (95% CI)	*p*	HR (95% CI)	*p*
CNSIPI scores ≥ 4	3.830 (1.948 ~ 7.527)	< 0.001	3.620 (1.662 ~ 7.887)	0.001
CNS recurrence	2.724 (1.321 ~ 5.617)	0.007	3.109 (1.494 ~ 6.469)	0.002

Abbreviations: CI, confidence interval; CNS, central nervous system; HR, hazard ratio; IPI, International Prognostic Index.

### 3.4 Toxicity of HD‐MTX Prophylaxis

In total, 46 patients completed 92 cycles of the HD‐MTX regimen. Of them, 49 cycles were administered on day 6 of the R‐CHOP regimen (the HD‐MTX day 6 group), whereas 43 cycles were initiated on day 10–14 following the R‐CHOP regimen (the HD‐MTX day 10–14 group). Additionally, 57 cycles of the R‐CHOP regimen, administered either before or following HD‐MTX treatment, served as the control group (the R‐CHOP group). Statistical analyses of the chemotherapy intervals among the three groups revealed significant differences (*p* < 0.001), with mean intervals of 30, 41, and 26 days for the HD‐MTX day 6, HD‐MTX day 10–14, and R‐CHOP groups, respectively.

The adverse events associated with different HD‐MTX chemotherapy groups are presented in Table 3. Notably, no statistically significant differences were observed in the incidence of hematological and nonhematological toxicities across all groups. Additionally, two patients experienced grade 3 acute renal injury following HD‐MTX administration, necessitating continuous renal replacement therapy. Nevertheless, no fatalities were reported to be associated with HD‐MTX chemotherapy.

**TABLE 3:** List related to different chemotherapy regimen groups.

Event	R‐CHOP group (*N* = 57)		HD‐MTX day 6 group (*N* = 49)		HD‐MTX day 10–14 group (*N* = 43)		*p* ^a^
	Any grade	Grade 3 or 4	Any grade	Grade 3 or 4	Any grade	Grade 3 or 4	
Hematologic toxicity							
Neutropenia	35 (61.4%)	32 (56.1%)	32 (67.4%)	29 (59.2%)	29 (67.5%)	26 (60.5%)	0.941
Anemia	33 (57.9%)	0	28 (57.1%)	0	27 (62.8%)	0	0.838
Thrombocytopenia	12 (21.1%)	0	11 (22.4%)	0	12 (27.9%)	0	0.710
Febrile neutropenia	4 (7.0%)		5 (8.2%)		4 (7.0%)		0.771
Nonhematologic toxicity							
Elevated hepatic transaminase	5 (8.8%)	0	7 (14.3%)	0	5 (11.6%)	0	0.672
Creatinine elevation	5 (8.8%)	0	8 (16.3%)	1 (2.2%)	9 (20.9%)	1 (2.3%)	0.221
Oral mucositis	8 (14.0%)	0	13 (26.5%)	0	13 (30.2%)	0	0.121

Abbreviations: HD, high‐dose; MTX, methotrexate; R‐CHOP, rituximab, cyclophosphamide, doxorubicin, vincristine, and prednisone.

^a^ Denotes the *p*‐value of any level of adverse event comparison.

## 4 Discussion

CNS recurrence in patients with DLBCL considerably exacerbates the disease status, and it is correlated with shorter survival and poor prognoses [3, 4, 5]. Therefore, early identification of high‐risk patients and implementation of effective preventive strategies is crucial to reduce CNS recurrence rates and improve patient survival outcomes. Although HD‐MTX therapy is commonly used for CNS prophylaxis in clinical practice, its efficacy regarding disease management remains controversial. Hence, this retrospective study evaluated HD‐MTX's prophylactic efficacy in patients with DLBCL presenting a high risk of CNS recurrence and compared CNS recurrence rates, PFS, and OS between patients receiving HD‐MTX and those who did not.

Overall, the findings of this study indicate that compared with the patients in the non‐HD‐MTX group, those in the HD‐MTX group exhibited a lower 2‐year CNS recurrence rate and a higher 2‐year PFS and OS rates. However, although differences in PFS and OS rates were statistically significant, the reduction in CNS recurrence rate was not. These results are consistent with those previously reported. For instance, Goldschmidt et al. analyzed 480 patients at high risk for CNS recurrence in Israel, of whom 130 (27%) received HD‐MTX therapy. Notably, HD‐MTX significantly improved both PFS and OS (*p* = 0.001) but could not reduce CNS relapse rates (HD‐MTX vs. non‐HD‐MTX was 6.9% vs. 6.3%, *p* = 0.97). They attributed the observed survival benefits to enhanced systemic disease control rather than the prevention of CNS relapse [14]. Notably, similar mechanisms may underlie the improved survival rates observed in the HD‐MTX group in the present study. Nevertheless, the potential contribution of reduced CNS recurrence to the observed survival benefit cannot be entirely dismissed, especially considering the downward trend of the CNS recurrence rate in the HD‐MTX group compared with that in the non‐HD‐MTX group. The multivariate analysis of PFS and OS revealed the incidence of CNS recurrence as the independent risk factor influencing patient survival. However, this needs to be further validated with a larger cohort, as this single‐center study may have an insufficient sample size for the low probability event of CNS recurrence.

Although the application of HD‐MTX in high‐risk populations for CNS recurrence may prolong survival or reduce disease progression, studies identifying specific subgroups affected by this treatment are lacking. Herein, the results of subgroup analyses on PFS and OS revealed that patients with better chemotherapy tolerance (NCCN‐IPI: 0–5; age ≤ 60 years; sex: male; and absence of B symptoms) or those at higher risk of CNS recurrence (CNS‐IPI ≥ 4) experienced more significant improvements in PFS and OS. Furthermore, patients who did not undergo ASCT showed greater survival benefits from HD‐MTX treatment, which may be attributed to its systemic control mimicking ASCT consolidation or overlapping CNS prophylaxis effects from ASCT conditioning regimens. Patients with GCB‐subtype DLBCL presented superior survival outcomes, which were associated with higher rates of double‐hit lymphoma in this subgroup (17.2% in GCB vs. 5.1% in non‐GCB, *p* = 0.022). Reportedly, the clinical characteristics of highly aggressive behavior and increased CNS recurrence rate in double‐hit lymphoma may result in it benefiting from HD‐MTX chemotherapy and effective CNS prophylaxis.

CNS recurrence is reported to predominantly occur in the early stages (median time for recurrence: 6–7 months) [4, 17]. Herein, the median TT‐CNS was 5.5 months, which is consistent with the abovementioned range. Consequently, many medical centers have incorporated intercalated HD‐MTX (i‐HD‐MTX) during the early cycles of the R‐CHOP regimen to prevent early CNS recurrence. However, Wilson et al. reported no evidence that completing the R‐CHOP regimen increased the risk of CNS recurrence compared with the i‐HD‐MTX regimen [18]. To the best of our knowledge, the largest study to date on HD‐MTX‐related adverse reactions is a retrospective study involving 258 patients performed in the United States. The patients were divided into the HD‐MTX (*n* = 128) and non‐HD‐MTX (*n* = 130) groups. Notably, the HD‐MTX group exhibited a higher incidence of grade ≥ 3 oral mucositis and increased alanine aminotransferase levels. Additionally, delays or dose reductions in the R‐CHOP regimen were more frequent in the HD‐MTX group compared with those in the non‐HD‐MTX group (31.2% vs. 16.9%) [19]. Delays in chemotherapy administration can adversely affect treatment efficacy. Recently, Song et al. conducted a multicenter retrospective clinical study on the impact of chemotherapy delays on prognosis in pediatric Burkitt lymphoma. They showed that children experiencing chemotherapy delays exceeding 7 days presented significantly lower 3‐year OS rates compared with those with delays of ≤ 7 days [20]. Consequently, it was recommended that HD‐MTX chemotherapy‐associated delays should be restricted to ≤ 7 days whenever feasible to mitigate treatment delay‐induced negative effects on therapeutic efficacy and patient prognosis.

Herein, the chemotherapy intervals of two HD‐MTX regimens (HD‐MTX day 6 vs. HD‐MTX day 10–14) and R‐CHOP were compared. Although both HD‐MTX regimens had chemotherapy delays, owing to the shorter delay, the HD‐MTX day 6 regimen presented superior results to the conventional HD‐MTX day 10–14 regimen. Regarding hematologic and nonhematologic toxicities, both HD‐MTX regimens showed no statistically significant differences compared to the R‐CHOP‐alone regimen. However, the incidences of high creatinine levels and oral mucositis were significantly higher with HD‐MTX regimens. These results are consistent with those previously reported.

This study has some limitations. First, the small sample size, coupled with constraints inherent in nonrandom observational and regression analyses, may have resulted in imbalanced baseline characteristics across different groups. Notably, the mean baseline age was significantly lower in the HD‐MTX group compared with that in the non‐HD‐MTX group. Typically, age is regarded as a high‐risk prognostic factor in lymphoma; however, in the present cohort of patients at high risk for CNS recurrence, age showed no association with survival outcomes in either univariate or multivariate prognostic analyses. Second, baseline CNS evaluation may have influenced the study results. Herein, baseline CNS assessment was not mandatory for all patients and was conducted only for those with a CNS‐IPI score of ≥ 4. Moreover, more sensitive detection methods for CNS involvement, such as measuring chemokine (C‐X‐C motif) ligand 13 and interleukin‐10 levels in the CSF, were not implemented clinically. Hence, patients experiencing early CNS relapse may not have been identified before initiating chemotherapy, either because of missed detection or insufficient sensitivity of present detection methods. Bennett et al. [15] reported the real‐world incidence of CNS relapse following baseline CSF analysis, which was conducted to rule out asymptomatic leptomeningeal involvement in patients newly diagnosed with high‐risk DLBCL. They showed that among patients with CNS involvement (indicated by a positive baseline CSF analysis), the incidence of CNS disease events over 5 years was 44.4%, compared with 5.6% for patients without CNS involvement (indicated by a negative baseline CSF analysis).

In this study, definitive results regarding the CNS prophylactic efficacy of HD‐MTX could not be achieved; nevertheless, the findings show the promising clinical viability of the HD‐MTX administration regimen—specifically the HD‐MTX day 6 regimen. This regimen effectively reduced the typical HD‐MTX‐associated chemotherapy delays. However, owing to the limited sample size and baseline imbalances, statistical analyses could not ascertain the optimal number of HD‐MTX cycles. Future studies need to focus on validating the HD‐MTX day 6 regimen and determining the optimal number of treatment cycles.

Altogether, the role of HD‐MTX in preventing CNS recurrence could not be conclusively determined due to the lack of robust evidence. However, the intravenous HD‐MTX day 6 regimen showed notable therapeutic potential for patients who can tolerate the treatment, such as younger individuals and those at NCCN‐IPI low or intermediate risk. Despite lacking results, this study serves as a valuable reference for future studies on HD‐MTX treatment and for decision‐makers to offer guidance on clinical treatment decisions.

## Author Contributions

**Yanli Wang:** conceptualization, methodology, funding acquisition, writing – original draft, project administration. **Xiaolian Wen:** investigation, data curation, resources, writing – review and editing. **Tao Guan:** data curation, methodology, resources, investigation. **Hongwei Zhang:** project administration, visualization, validation. **Wei'e Han:** resources, supervision, data curation. **Min Bai:** data curation, supervision, resources, investigation. **Xiaolan Liu:** resources, supervision, data curation, project administration. **Min Zhang:** data curation, supervision, resources, methodology. **Liping Su:** conceptualization, resources, data curation. **Weihua Zhang:** conceptualization, methodology, resources, writing – original draft.

## Ethics Statement

The Ethics Committee of the Shanxi Medical University Affiliated Cancer Hospital (Shanxi Provincial Cancer Hospital) which is registered as Institutional Review Board at the National Medical Research Registration and Filing Information System (https://www.medicalresearch.org.cn) approved the evaluation presented here (KY2023080).

## Conflicts of Interest

The authors declare no conflicts of interest.

## Data Availability

The datasets used and analyzed during the current study available from the corresponding author on reasonable request.
